# High flow conditions mediate damaging impacts of sub-lethal thermal stress on corals’ endosymbiotic algae

**DOI:** 10.1093/conphys/coab046

**Published:** 2021-06-24

**Authors:** C E Page, W Leggat, S F Heron, A J Fordyce, T D Ainsworth

**Affiliations:** 1 Life Sciences, Imperial College, Exhibition Road, London SW7 2AZ, UK; 2 School of Biological, Earth and Environmental Sciences, UNSW, Kensington, High St, New South Wales 2033, Australia; 3 School of Environmental and Life Sciences, University of Newcastle, University Dr, Callaghan, New South Wales 2308, Australia; 4 Physics and Marine Geophysical Laboratory, College of Science and Engineering, James Cook University, James Cook Dr, Townsville, Queensland 4811, Australia; 5 NOAA Coral Reef Watch, College Park, MD 20740, USA

**Keywords:** Bio-physical, climate change, coral bleaching, flow, interactions, thermal stress

## Abstract

The effects of thermal anomalies on tropical coral endosymbiosis can be mediated by a range of environmental factors, which in turn ultimately influence coral health and survival. One such factor is the water flow conditions over coral reefs and corals. Although the physiological benefits of living under high water flow are well known, there remains a lack of conclusive experimental evidence characterizing how flow mitigates thermal stress responses in corals. Here we use *in situ* measurements of flow in a variety of reef habitats to constrain the importance of flow speeds on the endosymbiosis of an important reef building species under different thermal regimes. Under high flow speeds (0.15 m s^−1^) and thermal stress, coral endosymbionts retained photosynthetic function and recovery capacity for longer compared to low flow conditions (0.03 m s^−1^). We hypothesize that this may be due to increased rates of mass transfer of key metabolites under higher flow, putatively allowing corals to maintain photosynthetic efficiency for longer. We also identified a positive interactive effect between high flow and a pre-stress, sub-lethal pulse in temperature. While higher flow may delay the onset of photosynthetic stress, it does not appear to confer long-term protection; sustained exposure to thermal stress (eDHW accumulation equivalent to 4.9°C weeks) eventually overwhelmed the coral meta-organism as evidenced by eventual declines in photo-physiological function and endosymbiont densities. Investigating flow patterns at the scale of metres within the context of these physiological impacts can reveal interesting avenues for coral reef management. This study increases our understanding of the effects of water flow on coral reef health in an era of climate change and highlights the potential to learn from existing beneficial bio-physical interactions for the effective preservation of coral reefs into the future.

## Teaser

Lay summary

High flow speeds can mediate damaging impacts of sub-lethal thermal stress on a branching coral species, showing increased levels of endosymbiont photosystem health compared to corals under low flow conditions. However, eventual declines in physiology and no difference in bleaching under high and low flow conditions indicate that eventually thermal stress may overwhelm positive impacts of flow.

## Introduction

Tropical coral reefs are invaluable in their ability to support marine biodiversity (Reaka-Kudla, 1997; Hughes *et al.*, 2002), provide resources to coastal communities (Costanza *et al.*, 1997; Woodhead *et al.*, 2019) and absorb energy protecting large areas of coastline (Harris *et al.*, 2018; Osorio-Cano *et al.*, 2019). Underlying this remarkable capacity for ecosystem function is a symbiotic partnership between scleractinian coral species and an algal dinoflagellate symbiont (of the family Symbiodinaceae; LaJeunesse *et al.*, 2018). The endosymbiont photosynthesizes and translocates fixed carbon in the form of sugars to the host, providing the coral with a majority of the carbon needed to grow and accrete calcium carbonate from the surrounding water column (Muscatine and Porter, 1977).

Yet, coral symbiosis is increasingly threatened by a myriad of disturbances from global (Hughes *et al.*, 2017; Hughes *et al.*, 2018) to local scales (McLean *et al.*, 2016; Wolff *et al.*, 2018). Exposure to environmental conditions to which a coral may not be locally acclimated can cause coral bleaching—a stress response resulting in the loss or reduction of endosymbionts and/or damage or loss of their associated pigments from the coral host cells (Gates *et al.*, 1992; Hoegh-Guldberg, 1999). For example, coral bleaching events are often linked with exposure to above average temperatures (i.e. thermal stress), exacerbated by high light conditions (and low wind speeds; Fordyce *et al.*, 2019). The impacts of climate change over reefs are varied: marine heatwaves may be severe causing widespread bleaching and mortality (Fordyce *et al.*, 2019; Leggat *et al.*, 2019), while more moderate events may cause bleaching with little to no mortality (Heron *et al.*, 2016). Warmer temperatures have also been linked to outbreaks of coral disease (Heron *et al.*, 2010; Randall and van Woesik, 2015) and the emergence of novel species interactions (Miranda *et al.*, 2018; Vergés *et al.*, 2019). As such the preservation of reefs requires effective management plans that can mitigate the cumulative negative impacts of both local and global stressors to conserve ecosystem function.

As a result of the rapid changes now being documented on coral reefs worldwide, there are a number of novel intervention actions that have been proposed (Bay *et al.*, 2019; Board, Ocean Studies, National Academies of Science Engineering and Medicine, 2019; Ainsworth *et al.*, 2020; Morrison *et al.*, 2020), including the development of coral cryobiology (Hagedorn and Carter, 2016), coral probiotics (Rosado *et al.*, 2019) and the potential engineering of ‘super corals’ (Camp *et al.*, 2018a; Camp *et al.*, 2018b; Buerger *et al.*, 2020). There has also been a push to use new technologies to more effectively monitor reefs (Bajjouk *et al.*, 2019; Calders *et al.*, 2019) and various restoration methods are now becoming more refined and scalable (Suggett *et al.*, 2019). These approaches are not without pitfalls but can provide innovative ways to preserve such critical ecosystems into the future. Similarly, there now is also the development of reef conservation approaches that aim to harness existing beneficial environmental and ecological interactions and utilize these to support coral health and survival (Halpern *et al.*, 2007; Timpane-Padgham *et al.*, 2017; Ladd *et al.*, 2018; Ainsworth *et al.*, 2020).

Importantly, reef environments are often highly heterogeneous in both abiotic and biotic interactions (Lenihan *et al.*, 2008; Guadayol *et al.*, 2014; Rogers *et al.*, 2016; Hoogenboom *et al.*, 2017). The interplay between these, referred to as ‘bio-physical’ interactions, ultimately has the capacity to affect the responses of colonies to sources of physiological stress (West and Salm, 2003; van Woesik *et al.*, 2005; Smith and Birkeland, 2007; Hoogenboom and Connolly, 2009; Schutter *et al.*, 2010; Davis *et al.*, 2011; Ainsworth *et al.*, 2016; DeCarlo *et al.*, 2017; Darling and Côté, 2018; Page *et al.*, 2019). Interestingly, the temperature regime that an individual coral experiences prior to surpassing its thermal bleaching threshold can also influence physiological and therefore ecological outcomes (Ainsworth *et al.*, 2008; Ainsworth *et al.*, 2016). Even week-long increases in mild stress with sub-lethal effects during a conditioning period can play a protective role, directly causing a reduction in magnitude of subsequent stress responses such as apoptosis (Bellantuono *et al.*, 2012; Ainsworth *et al.*, 2016).

Water flow conditions over reefs have been shown to mediate the physiological outcome of thermal stress on coral health (Nakamura and van Woesik, 2001; McClanahan *et al.*, 2005; Skirving *et al.*, 2006; Smith and Birkeland, 2007; DeCarlo *et al.*, 2017; Wolanski *et al.*, 2017; Page *et al.*, 2019). The relationship between bleaching patterns and flow was recognized after the 1998 bleaching event in Japan where coral survival was positively associated with areas of higher flow (Nakamura and van Woesik, 2001). Similarly, the survival of corals on offshore islands during the 2002 bleaching event in the Arabian Gulf was linked to higher levels of water motion compared to conditions further inshore (Riegl, 2003). High water flow can enhance primary production, dark respiration and particle capture in corals, thereby positively affecting growth (Patterson *et al.*, 1991; Sebens and Johnson, 1991; Finelli *et al.*, 2006; Finelli *et al.*, 2007; Mass *et al.*, 2010; Osinga *et al.*, 2017). In contrast, low flow speeds (<0.03 ms^−1^) have been recorded as contributing to ‘extreme’ bleaching conditions, leading to rapid coral mortality over reefs (DeCarlo *et al.*, 2017; Baird *et al.*, 2018).

The relationship between water flow and coral function at the colony level is hypothesized to be linked to increased flow speeds creating thinner boundary layers, which increase mass transfer of gases and metabolites, increasing the rates of physiological processes (Patterson, 1992; Falter *et al.*, 2005; Carpenter and Williams, 2007; Falter *et al.*, 2007; van Woesik *et al.*, 2012; Page *et al.*, 2019). Specifically, under thermal stress it has been suggested that higher flow speeds may positively impact coral function through reducing heat-induced oxidative stress (Nakamura and van Woesik, 2001; Nakamura, 2010). Although experimental studies to date offer support for the beneficial impacts of high flow on coral responses to general environmental stress (Nakamura and van Woesik, 2001; Nakamura *et al.*, 2003; Nakamura *et al.*, 2005), limited experimental comparisons and detail of the responses measured, coupled with exposure to extreme treatments (e.g. high flow conditions of 0.5 to 0.7 m s^−1^ and 95% irradiance; Nakamura and van Woesik, 2001), result in insufficient evidence to conclude the extent to which flow may be causing differential responses to thermal stress. In this paper, we look to assess the impacts of flow on coral function and in doing so provide experimental evidence to characterize how flow may modulate thermal stress responses in corals. Resolving the biological consequences of flow at reef and within-reef scales is important to comprehensively understand how flow affects resistance of corals to thermal stress.

Here we investigate whether water flow speeds (high and low) can mediate the impacts of multiple climate change-related thermal regimes on a thermally susceptible coral species, *Acropora aspera* (Loya *et al.*, 2001; van Woesik *et al.*, 2011; Nitschke *et al.*, 2018). Using Heron Island on the Southern Great Barrier Reef (GBR) as a case study, we contextualized experimental flow simulations through characterizing the flow conditions to which corals are exposed within two reef habitats: the flat intertidal region of reef lagoon (flat) and the sloping reef towards a deep water channel (slope). To investigate the impacts of water flow on an initial stress response through to bleaching, we exposed fragments of *A. aspera* collected from the reef flat to a range of thermal stress regimes over two experimental periods. Specifically, we designed three temperature treatments based on the thermal threshold for Heron Island Reef Flat: a sub-bleaching trajectory, a sub-bleaching trajectory with a pre-stress pulse in temperature (Ainsworth *et al.*, 2016) and a bleaching trajectory involving a direct increase in temperature to the thermal threshold for Heron Island Reef Flat (34°C). This relatively high bleaching threshold is owed to the extremely variable environment to which corals are acclimatized to (Kline *et al.*, 2012; Ruiz-Jones and Palumbi, 2017). The three temperature treatments were achieved through manipulation of the rate of temperature increase and maximum daily temperatures, and the stress responses of the coral host and their endosymbiotic algae were measured using photophysiology and quantification of endosymbiont densities.

## Materials and methods

**Figure 1:**
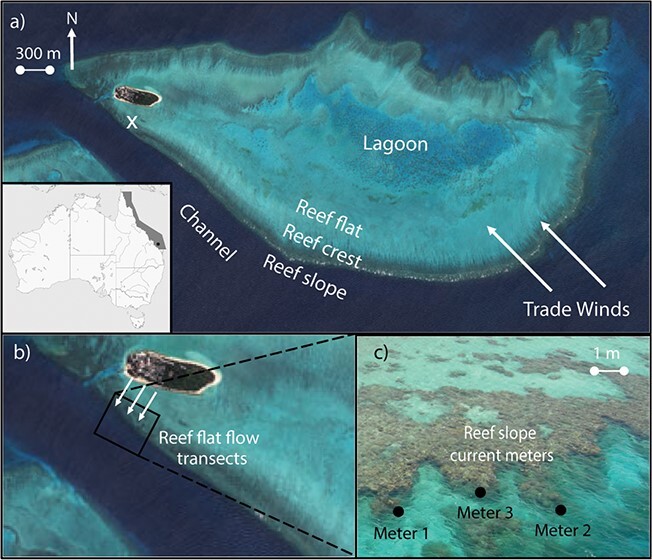
**(a)** Heron Island (23°26.1871″S, 151°54.3023″E) is situated on the Southern GBR. The position of Heron Island is marked by a black circle. Different reef zones are shown. A white x marks coral collection from reef flat. **(b)** Reef flat flow transects. Flow speed was measured along three transects at five points spaced by 10 m using an ADV held at the average height of the surrounding coral benthos (~0.2 m). **(c)** Three current metres were deployed at Coral Gardens (23°26.698″S, 151°54.533″E) adjacent to where the reef flat measurements of flow velocity were measured, and corals were collected. Metres 1 and 2 were placed on exposed areas of the reef, whereas flow metre 3 was placed in a more sheltered position. Images provided by DigitalGlobe.

Research was conducted on Heron Island Reef (23° 44′23′′S, 151° 91′48′′E), a lagoonal platform reef located 80 km northeast of Gladstone near the Tropic of Capricorn (Fig. 1). The benthic cover of Heron Island Reef is characterized by distinct ecological zones of differing coral cover including the lagoon, reef flat, reef crest and reef slope (Fig. 1). The wind regime of Heron Reef is dominated by the south-easterly trade winds, with more variable winds during summer (MacKellar and McGowan, 2010). Tides at Heron Reef are semi-diurnal, with spring and neap tidal ranges of 2.28 m and 1.09 m, respectively (Chen and Krol, 2004). At low tide, water depth over much of the reef flat is 0.3–1 m, while in the deeper part of the lagoon it averages 3.5 m (Chen and Krol, 2004).

### *In situ* measurements of flow conditions

#### Reef flat

To measure the range of flow conditions corals experience on the southwestern reef flat of Heron Island (the site of coral sample collection), measurements of water speed were taken over three transects running from the beach to the reef slope, each 60 m in length (Fig. 1b). Six measurements of average flow speed (recorded over 3 min each) were taken along each transect at 10 m increments using the FlowTracker 1 (SonTek) Acoustic Doppler Velocimeter (ADV, measuring range 0.001 m s^−1^–4.5 m s^−1^ and accurate to 1% of the measured velocity in a 1-s sample). Measurements were taken over 3 days (8 February 2019, 9 February 2019 and 11 February 2019) at 1 h after the highest tide (the second half of ebb tide, between high and low tide; Roberts and Suhayda, 1983). During each reading, the ADV was held stationary in the water column through attachment to a pole, at approximately the mean height of the surrounding benthos (~0.2 m)—this was to ensure that measurements of speed were minimally impacted by depth. Following manufacturer’s instruction, the signalling arm of the ADV probe was placed so that it faced the primary flow direction.

#### Reef slope

To measure the range of flow conditions on the reef slope, three Marotte HS drag-tilt current metres (http://www.marinegeophysics.com.au/current-meter/) were deployed at Coral Gardens, a site immediately adjacent to where flow conditions were measured on the reef flat (measuring range 0.05 m s^−1^—1.2 m s^−1^, with an expected error of ±0.05 m s^−1^ at the expected flow speeds of < 0.10 m s^−1^). The topography at the site made it possible to measure the range of flow conditions in sheltered and exposed habitats, capturing some of the variation that slope corals experience. Two metres were deployed on exposed spurs of the canyon, while the third was placed in a sheltered groove behind a coral bommie (Fig. 1c). Metres were deployed through attachment to star pickets hammered into rubble substrate. Metres were attached to the pickets using cable ties following manufacturer’s instructions. Metres were placed at the same height as the coral canopy in the water column, with the tallest part of the metre ~1 m from the seafloor, sitting at a depth of ~ 3 m. This area of the reef slope is not exposed at high tide. Current metres took measures of flow speed (m s^−1^), direction (degrees) and temperature (°C) every minute for a total of 23 days. To visualize how variation in tide and wind speed may impact flow conditions, daily tide height predictions and 10-min averaged wind speeds were obtained from the Australian Government Bureau of Meteorology for the Gladstone area and Heron Island Research Station for the time that the metres were deployed.

### Experimental design

#### Coral collection

For both experimental periods, fragments (single branches of maximum branch length 7 cm) of *A. aspera* (Experiment 1, *n* = 54; Experiment 2, *n* = 36) were collected over two consecutive days in each of March 2018 and February 2019 within the scientific zone of the reef flat (see Fig. 1a). *Acropora aspera* forms expansive branching thickets, making it difficult to distinguish individual colonies and genotypes. Therefore, sampling took place at distinct coral patches separated by at least 15 m to maximize genetic diversity and limit the risk of using genetic clones as replicates (Combosch and Vollmer, 2011). At each patch, no more than 5% of the total patch was collected. Fragments were randomly assigned to high volume (600 l) experimental mesocosm tanks under ambient flow-through conditions ((Fordyce *et al.*, 2017) pH 7.980–8.020; conductivity of 53–54 μSm^−1^; temperature 26–30°C; and PAR 0–3875 μmol m^−2^ s^−1^) where they were held upright in test tube racks for a period of up to 20 days, to allow recovery from collection. The length of acclimation period varied for each experiment and flow conditions were also set up during the acclimation period before thermal ramping commenced on different days for each experiment; see Sections 2.3.1 and 2.4.1 for details. Recovery was evaluated based on re-growth of coral tissue over the collection wound. Fragments that did not recover were not included within experiments.

#### Experimental system

**Figure 2:**
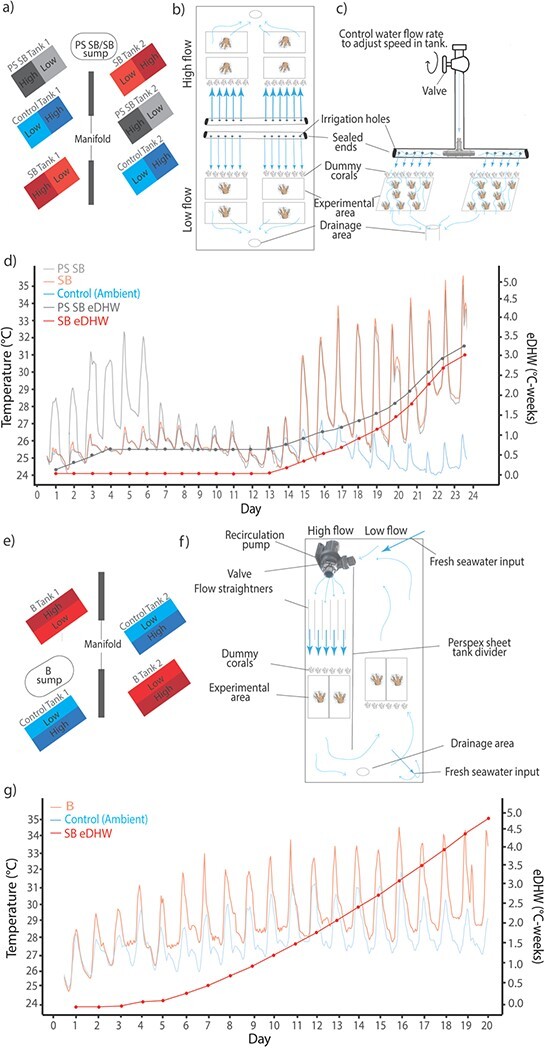
**(a)** Experimental layout of tanks, Experiment 1. Flow treatments are nested within thermal treatment. PS SB/SB sump is the sump used to heat the PS SB (for the pre-stress stage up to ~32°C, and for the stress treatment up to ~ 34°C), and SB thermal treatment tanks (for the thermal stress period up to ~ 34°C). **(b)** Diagram of the T-Pipe flow set-up used in Experiment 1. Identical irrigation T-pipes were used for each flow treatment to create target high and low flow speeds of ~0.15 m s^−1^ and ~0.03 m s^−1^. **(c)** Diagram showing a cross-section of the flow set-up used in Experiment 1. High and low flow treatments were created through adjusting of the water into the T-pipe through the valve position. Dummy coral skeletons acted to disturb laminar flow, inducing turbulence and ensuring similar flow conditions experienced by all coral fragments in the experimental area. **(d)** Experiment 1: sub-bleaching thermal stress experimental period (24 days, 12 April–5 May 2018). Plot displays the hourly average temperature across all treatment tanks. The blue line is the control (ambient) treatment, reflecting temperatures experienced by corals on Heron Island reef flat. The light red line is SB thermal treatment, and the light grey line shows average temperatures in PS SB treatments. **(e)** Experimental layout of tanks, Experiment 2. Flow treatments are nested within thermal treatment. B sump is used to heat the B thermal treatment (gradual ramping up until ~34°C daily maxima). Thermal stress accumulation was calculated for thermal treatments (PS SB, SB) as eDHW and was shown as dark grey and red lines. **(f)** Diagram of the recirculation pump flow set-up used in Experiment 2. The recirculation pump was used to move water through flow straighteners across the experimental area, creating target high flow speeds of ~0.15 m s^−1^. Target flow speeds were created through adjustment of flow rate valve on the pump. Recirculation of water around the tank and loss of water at the drainage point created low flow speeds of ~0.03 m s^−1^ on the opposite side of the tank. New saltwater input was fed into the tanks adjacent to the recirculation pump input and before water moved over the low flow side of the tank. **(g)** Experiment 2: thermal stress experimental period (20 days, 4 March–24 March 2019). Plot displays the hourly average temperature across all treatment tanks. The blue line is the control (ambient) treatment, reflecting temperatures experienced by corals on Heron Island Reef flat. The light red line shows temperatures in the B thermal treatment. Thermal stress accumulation was calculated for thermal treatments B as eDHW and is shown by a dark red line.

Experiments were performed in a semi-closed system, where unfiltered sea water pumped from Heron Island Reef flat was fed continuously into a 1000 l sump covered in opaque tarps (Fig. 2a and e). Corals were housed in 600 l outdoor mesocosm tanks, consisting of a working area of 0.63 m (width) × 1.71 m (length) × 0.25 m (height). Corals in the mesocosm tanks were exposed to daily temperature variations of 4–5°C (Fig. 2d and g), similar to ambient conditions on the reef flat. On some days, external environmental conditions (i.e. high solar irradiance) led to tanks reaching peak temperatures higher than target temperatures for small periods of time around mid-day.

The experimental design was nested with respect to flow treatment levels, where each tank was split in half and flow treatments (high and low) were set up in each half, respectively. For each temperature treatment (heat treatments and control), there were two tank replicates. In total, three temperature treatments were applied: two (sub-bleaching and a pre-stress sub-bleaching trajectory) in the first experimental period, and one (bleaching) during the second experimental period. During each experimental period, a control treatment received ambient water (~27°C) directly from the reef flat. Tanks were positioned to reduce bias arising from external environment effects (Fig. 2a and e) such as shading from surrounding buildings. Coral fragments were randomly assigned to each of the temperature and flow treatments, respectively. Temperatures in heat treatments were increased gradually at ~1°C/day to prevent heat shock, until target temperatures were achieved. Sumps were plumbed directly to the thermal treatment tanks and directly heated with aquarium heaters (Aquasonic) in accordance with respective temperature stress profiles. HOBO pendant temperature loggers recorded seawater temperatures every minute in all tanks. Two Odyssey photosynthetic active radiation (PAR) recorders (Dataflow Systems Limited, Christchurch New Zealand) were placed randomly across the experimental treatments to capture in-tank irradiance levels every 30 s (Fig. S1). See Fig. 2 for a diagram of experimental tank design.

#### Calculation of total heat accumulation in treatments: eDHWs

To track the accumulation of temperature stress to which corals were exposed under the different thermal treatments during the experimental period, degree heating weeks (DHWs) were calculated for each day following methods outlined by Gierz *et al.* (2020) (see Equation 1). Used traditionally as a remote sensing metric, Coral Reef Watch’s DHW product is commonly used to predict the timing and intensity of coral bleaching. It is calculated through integrating the instantaneous bleaching heat stress during the most recent 12-week period (Liu *et al.*, 2014). Instantaneous heat stress is measured by Coral Bleaching HotSpot as the positive anomaly (HotSpot’s above 1°C) above the long-term average temperature of the climatologically warmest month at each location (the mean monthly maximum, MMM). Positive values indicate thermal stress, where 4°C weeks is seen as an alert for significant bleaching, and 8°C weeks indicates extreme warming with likely severe bleaching (Liu *et al.*, 2014). Through altering this method, we are able to calculate a comparable metric, experimental DHWs (eDHWs), for use in experimental systems. eDHWs are a measure of heat accumulation calculated by taking the cumulative difference between the average daily observed water temperature in treatment tanks (when the difference is greater than or equal to 1), and the MMM for Heron Island (27.3°C,), divided by seven. Code for calculation in R is provided in the online data and code repository (https://charlotteepage.github.io/Flow_effects_thermal_stress_A.aspera/).

(1) \begin{align*} eDHW\!=\!\sum \frac{if\ \Big( daily\ average\ temperature- \!MMM\Big)\!\ge\! 1{}^{\circ}\mathrm{C}\ }{7} \end{align*}

### Experiment 1: sub-bleaching thermal stress

#### Flow speeds

Each mesocosm was divided across the tank into two halves by an irrigation system, which maintained low (~0.03 m s ^−1^) and high (~0.15 m s ^−1^) flow conditions, respectively, in each half tank. The irrigation system passed water from an overhead tap, into the tank through a T-junction piping structure (Fig. 2c). Five millimetre diameter holes, parallel to the length of the tank, were drilled into each arm of the T-pipe, approximately 0.07 m apart. Two T-pipes were placed in each tank so that water was being distributed at a height approximately at the mid-height of the coral branches (0.045 m), setting up high and low flow conditions, respectively, in each end of the raceway (Fig. 2b). Corals (*n* = 15) were placed 0.2 m away from the T-pipe. Test tube racks holding corals were placed in front of each arm of the T-pipe. To ensure that all corals were receiving similar flow conditions, a row of coral skeletons (termed ‘dummy corals’) (*n* = 6) were placed in front of the holding racks (Fig. 2b and c). The dummy corals acted to break up the flow from the T-pipe, ensuring that the first row of corals did not receive differential and potentially higher flow conditions than the proceeding corals. In this way, the coral branches experienced the more turbulent flow conditions characteristic of shallow reefs (Monismith, 2006). Flow conditions were maintained through manually adjusting the outflow of water into the T-pipe (Fig. 2c). Water speed in each half raceway was measured throughout the experimental period (and adjusted accordingly), from distance–time data recorded by injecting non-toxic red coloured dye into the water column at the mid-height of the coral branches and documenting the time taken to reach the end of the experimental area through the analysis of video footage. Target flow speeds were established on Day 5 of the 14-day recovery period to allow corals to acclimate to their respective flow conditions prior to application of the thermal stress trajectories.

#### Temperature treatments

Following the 14-day recovery period, corals were exposed to thermal stress trajectories that replicated: (i) sub-bleaching thermal trajectory (Ainsworth *et al.*, 2008; Bonesso *et al.*, 2017) (labelled ‘SB’), and (ii) a trajectory of a pre-stress increase in temperature prior to an increase in temperature to sub-bleaching levels (labelled ‘PS SB’) identified by Ainsworth *et al.* (2016) (Fig. 2d).

i) Sub-bleaching temperature treatment (SB): corals experienced no pre-stress event and were initially kept at ambient SST (~26°C daily maxima) for 14 days, before a gradual increase up to the bleaching threshold (~34°C daily maxima) across 10 days (ramping rate of ~ 0.8°C day ^−1^). Temperatures were held at a daily maximum of ~ 34°C for 4 days (Fig. 2d).
ii) Pre-stress sub-bleaching temperature treatment (PS SB): corals were exposed to a gradual increase in temperature across a period of 6 days up to 2°C below the thermal bleaching threshold (~32°C daily maxima, ramping rate of ~ 1°C day^−1^), followed by a relaxation in temperature to ambient (~26°C daily maxima) for 8 days, and then a gradual increase up to the bleaching threshold (~34°C daily maxima) for 10 days (ramping rate of ~ 0.8°C day ^−1^). Temperatures were held at ~ 34°C for 4 days (Fig. 2d).

### Experiment 2: bleaching thermal stress simulation

#### Flow speed

Each tank was divided lengthwise into two halves by a Perspex sheet, creating a high (~0.15 m s^−1^) and low (~0.03 m s^−1^; control) flow side of each tank. Flow speeds in each half of the tank were created through the placement of a recirculation pump at the upstream end of the high flow side of the tank (Fig. 2f), pushing a controlled volume of water down that side of the tank. Speed was set through controlling the volume of water recirculated from the pump by manual manipulation of a valve. Flow straighteners placed 0.2 m in front of the pump acted to spread out the flow of water evenly in the water column. Water then moved past a row of dummy corals (*n* = 6) placed 0.2 m away from the flow straighteners and then past two test tube racks holding nine coral fragments each. At the end of the high flow experimental area, water moved towards a drainage point, here saltwater input replaced this lost water. Water recirculated from the drainage end of the tank back towards the recirculation pump, creating low flow speeds on the other side of the tank. Here, water again moved past a row of dummy corals (*n* = 6) and then past two test tube racks holding nine coral fragments each. See Fig. 2 for a diagram illustrating the setup. Flow conditions were set up on Day 10 of the 20-day recovery period to allow corals to acclimate to their respective flow conditions prior to application of the thermal stress trajectories. Water speed in each side of the tank was measured instantaneously throughout the experimental period using a FlowTracker 1 (SonTek) ADV, capable of measuring 2D water speed. Measurements were taken at the beginning, middle and end of experimental period, half-way across the experimental area (0.2 m) to determine the average flow speeds experienced by corals. Each measurement was taken at 0.07 m in the water column (the same height as the fragments) and averaged over three minutes.

#### Temperature treatments

Following the 20-day recovery period, corals in heat treatment tanks were exposed to the bleaching temperature trajectory (labelled ‘B’) (Fig. 2g).

iii) Bleaching temperature treatment (B): corals were exposed to a gradual increase in temperature across a period of 10 days up to the bleaching threshold (~34°C daily maxima, a ramping rate of ~0.8°C day ^−1^). Corals were then exposed to ~34°C daily maxima for 10 days (see Fig. 2g).

### Measures of photophysiology and coral bleaching severity

#### PAM fluorometry of chlorophyll fluorescence

Chlorophyll fluorescence can be used to calculate a number of different photosynthetic metrics in a non-invasive way and consequently can be used as a proxy for the health of endosymbiont populations. Over the duration of both experiments maximum dark-adapted quantum yield (*Fv/Fm*) of chlorophyll a fluorescence was measured at least 30 min after sunset for dark adaption to assess the efficiency of photochemistry. All photochemical measurements were made using an imaging pulse-amplitude-modulated (PAM) fluorometer with MAXI head (Waltz, Effeltrich, Germany). The minimum fluorescence (*Fo*) was measured with a weak pulse of light, followed by saturating pulses of 2700 μmol m^−2^ s^−1^ of PAR for 0.8 s to establish the maximal fluorescence (*Fm*). Variable fluorescence yield (*Fm—Fo*) is then used to calculate the dark-adapted maximum quantum yield (*Fv/Fm*)*.* Fragments were immediately returned to mesocosms following measurement. In Experiment 1, measurements of quantum yield were taken on Days 3–24. In Experiment 2, measurements of quantum yield were taken on Days 1, 3 and 5–20.

In addition to quantum yield, induction and recovery curves were determined on coral fragments in Experiment 2 on Days 6, 12 and 15–20. As a more sensitive physiological measure than dark-adapted yield, this measurement examines the ability of the endosymbiont to acclimate to and recover from short-term light stress. This measurement was taken to record any physiological differences that may not be shown through typical quantum yield measurements. Specifically, the induction and recovery kinetic recording type on the PAM fluorometer (Waltz, Effeltrich, Germany) was used to examine the ability of endosymbiont PSII to dissipate excess light energy and recover from light stress. Following dark adaption for at least 30 min, minimum chlorophyll fluorescence (*Fo*) was determined using blue measuring light (Intensity 2) and maximum chlorophyll fluorescence (*Fm*) was determined by applying a pulse (0.72 s) of saturating light (Intensity 5, ~2800 μmol quanta m^−2^ s^−1^) allowing calculation of the dark-adapted maximal quantum yield of PSII (*Fv/Fm*). For the induction curve, actinic illumination (254 μmol quanta m^−2^ s^−1^, intensity 6) was switched on and 15 saturating pulses of PAR (~2800 μmol quanta m^−2^ s^−1^, Intensity 5, 0.72 s) were applied at 20 s intervals for 5 min. During the recovery phase, a further 16 saturation pulses were applied within a 7-min period without actinic illumination, where time between each pulse exponentially increased. Imaging-PAM fluorometry was then used to determine photo-kinetic parameters, such as the maximal quantum yield of PS II (*Fv/Fm*), and the effective quantum yield at the end of the induction. Light levels were measured using a LI-190SA Quantum Sensor with a LI-250A Light Meter (LI-COR^®^ Inc., NE, USA). Each coral fragment was immediately returned to its respective treatment following PAM measurements. At the end of the experiment, these fragments were returned to the reef flat.

#### Endosymbiont densities

To determine the effects of the different temperature trajectories and flow treatments on bleaching responses of *A. aspera*, endosymbiont densities were determined. Coral fragments (*n* = 3) were randomly sampled from each treatment at each time point (as described below). To determine whether the sub-bleaching temperature trajectories had caused significant reductions in levels of endosymbionts in Experiment 1, cell densities were determined before corals were exposed to the final increase in temperature stress on Day 13 of the experimental period and at the end of the experimental period (Day 24, Fig. 2g). To determine whether the bleaching temperature trajectory had caused significant reductions in levels of endosymbionts in Experiment 2, cell densities were determined before any temperature stress had accumulated (Day 1) and at the end of the experimental period (Day 20, Fig. 2g).

Tissue was airbrushed from the surface of each coral fragment using 50 ml of 0.45 μm filtered seawater. Following three washes (centrifuged at 3856 × g, 4°C for 5 min) of the algal pellet to remove mucous and coral tissue, the pellet was suspended in 10 ml of filtered sea water and aliquots were counted in triplicate per sample using an improved Neubauer haemocytometer. The 3D surface area of each fragment was calculated using a modified version of the wax method (Stimson and Kinzie, 1991). Wax dipping was conducted using paraffin wax at 65°C and each coral, or calibration object, was weighed prior to dipping and then dipped for 2 s before being removed and rotated quickly in air to promote even wax coverage. Dipped corals or calibration objects were then allowed to stand for 5 min before being reweighed. The first wax layer seals the internal pores of the skeleton. This process was then repeated so that the coral is dipped in a second layer of wax. This second layer covers only the surface area of the fragment. Using calibration objects of known surface area, a relationship was formed between the weight of the second wax layer and the actual surface area of the object, which was then used to calculate the surface area of the skeletons (Stimson and Kinzie, 1991).

### Statistical analysis

Summary statistics were calculated for *in situ* measures of flow conditions on the reef flat and reef slope. For the reef slope, summary statistics were calculated for each metre over the 23-day period. To further investigate how tidal patterns could be influencing flow speeds during this period, summary statistics were also calculated for two 5-day periods centred around moon phases forcing spring (17–21 April 2019) and neap tides (25–29 April 2019). An additional analysis was conducted on the reef slope flow data to investigate variation between current metres and is presented in the (see S1). Flow speeds measured in tanks throughout the experimental periods were averaged to quantify mean flow speeds of high and low flow treatments.

A statistical analysis on photo-physiological data and endosymbiont densities for each species were performed in R (RStudioTeam, 2019). Once data were checked for normality and homogeneity of variance, a mixed effects model was used to analyse the effect of temperature treatment and flow condition on quantum yields and endosymbiont densities for each experiment. Treatment and day were classified as fixed effects, while coral ID was treated as a random effect nested within each tank replicate. Significant differences were examined within each day using a sequential Bonferroni *post hoc* test.

All code for statistical analysis and data can be found on the online GitHub repository (https://charlotteepage.github.io/Flow_effects_thermal_stress_A.aspera/).

## Results

### *In situ* measurements of flow conditions over Heron Island reef

#### Reef flat

Average flow conditions over Heron Island Reef flat measured halfway between high and low tide over consecutive days showed mean flow speeds across all transects of 0.123 m s^−1^ ± 0.061 m s^−1^ (see Table 1) and upper and lower confidence intervals of 0.16 ms^−1^ and 0.093 ms^−1^, respectively. Highest flow conditions were measured 10 m away from the beach, where the reef habitat is characterized as an exposed sandy channel with low coral cover through which lagoonal waters drain at the dropping of the tide. Speed generally decreased from the beach towards the reef slope (Fig. S2).

**Table 1:** Summary statistics for *in situ* flow conditions measured on Heron Island reef flat and reef slope environments

	Minimum (ms^−1^)	Maximum (m s^−1^)	Average (m s^−1^)	Average (m s^−1^)	Average (m s^−1^)
Reef flat: measured using an ADV					
	0.027	0.365	0.123 ± 0.061		
Reef slope: measured using Marotte HS drag-tilt current metres					
Metre	Total period (11 April 2019–04 May 2019)			Spring (17 April 2019–21 April 2019)	Neap (25 April 2019–29 April 2019)
1	0.0003	0.2293	0.0830 ± 0.00023	0.0969 ± 0.0006	0.0511 ± 0.0004
2	0.0004	0.2018	0.0870 ± 0.00016	0.0958 ± 0.0004	0.0668 ± 0.0004
3	0.0003	0.1980	0.0675 ± 0.00018	0.0811 ± 0.0005	0.0436 ± 0.0003

Averages are presented with ± standard errors.

**Table 2:** Flow treatments measured in replicate tanks over the experimental period

Flow treatment	Average (m s^−1^)
Experiment 1: sub-bleaching thermal stress simulation	
Measured using distance-time data from the movement of dye	
High	0.16 ± 0.033
Low	0.04 ± 0.003
Experiment 2: bleaching thermal stress simulation	
Measured using an ADV	
High	0.15 ± 0.004
Low	0.02 ± 0.001

Flow measurements from Experiment 1 were taken using distance-time data of the movement of dye over the experimental area. Measurements were taken across each replicate tank throughout the experiment (high *n* = 18, low *n* = 17). Flow measurements from Experiment 2 were taken using an ADV. Measurements were taken across each replicate tank throughout the experiment (high *n* = 12, low *n* = 12).

#### Reef slope

The longest period of time for which Marotte current metres simultaneously logged was 11 April 2019–04 May 2019, a total of 23 days (Fig.  S3). Average current speeds measured at each metre through the entire period ranged between 0.068 and 0.087 m s^−1^, with maximum speed at current metre 1 of 0.23 m s^−1^ (Table 1). The lunar phase was used to identify the centre of two 5-day periods encompassing spring (17–21 April 2019) and neap tides (25–29 April 2019) (see Table 1) during which spring-tide average speeds were up to twice those in neap periods (Table 1 and Figs S3 and S4). Results of the additional analysis conducted on the reef slope flow data are presented in the Supplemental Materials of this manuscript (see S2 and Figs  S4–S7).

### Experiment 1: sub-bleaching thermal stress simulation

Over the 24-day experimental period, fragments of *A. aspera* were exposed to two temperature treatments and average high and low flow speeds of 0.16 ± 0.033 and 0.04 ± 0.003 (Table 2). The PS SB exposed corals to an increase in temperature up to 32°C daily maxima (a sub-lethal pulse in temperature) with a ramping rate of 0.7°C day^−1^, reaching 0.57°C weeks prior to relaxing at ambient levels for 8 days (~26°C), and then an initial (first 4 days) ramping rate of 0.6°C day^−1^ until the bleaching threshold was reached (~34°C daily maxima) for 6 days, accumulating a total of 3.19°C weeks by the end of the experimental period (Fig. 2d). The SB treatment exposed corals to the identical final increase from ambient levels (~26°C daily maxima) to the bleaching threshold (~34°C daily maxima) held for 6 days, accumulating a total eDHW of 3.03°C weeks by the end of the experimental period (Fig. 2d). These temperature treatments led to sub-bleaching levels of thermal stress, with limited signs of paling or mucous sloughing when exposed to daily peak temperatures (Fig. 2d).

#### PAM fluorometry of chlorophyll fluorescence

**Figure 3:**
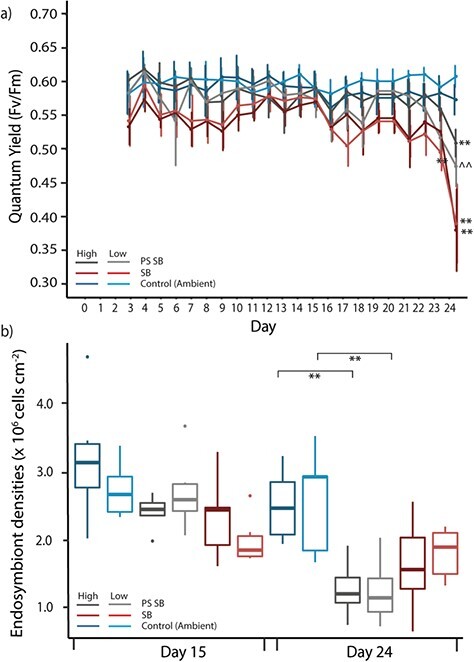
**(a)** Photophysiological measures of quantum yield during Experiment 1: sub-bleaching experiment. Mean ± SE are shown for each treatment beginning from Day 4 of the experimental period. Asterisks indicate a significant difference between the treatment and its respective control (*P < 0.05, **P < 0.01), while ^^ indicates significant difference between PS SB high flow and SB high flow corals (*P* < 0.01). **(b)** Endosymbiont densities on Day 13 and Day 24 of the sub-bleaching experiment. Box plots upper and lower lines correspond to the first and third quartiles and whiskers represent the minimum and maximum values. Points indicate outliers. Asterisks indicate significant differences between heat treatments and their respective control on each day (*P < 0.05, **P < 0.01). On both plots, blue, grey and red colours signify the ambient, PS BS and BS temperature treatments, while the darker shade within a temperature treatment represent high flow and lighter shades are low flow conditions.

Over the duration of Experiment 1, the quantum yield (*Fv/Fm*) of corals in ambient conditions remained high and constant. Eventual declines in yield for high and low flow heat treated corals (both PS SB and SB trajectories) were measured in the last two days of the experiment (Fig. 3). A mixed effects analysis of quantum yield shows a significant two-way interaction between day and temperature trajectories [*F*_(42,655)_ = 4.016, *P* < 0.01] and between flow and temperature trajectories [*F*_(2,655)_ = 4.674, *P* = 0.010] (Table S1). High flow, SB corals did not show declines in yield when compared to controls until Day 24 (*P* < 0.001), while corals exposed to high flow and PS SB thermal regime retain high quantum yields on Day 24. On Day 24, significant declines in yield were also recorded for PS SB low flow corals (*P* < 0.001), whereas fragments exposed to the SB heat treatment and low flow showed significant declines in yield a day earlier, on Day 23 (*P* = 0.023) (Fig. 3a).

#### Endosymbiont densities

Samples for endosymbiont densities were taken on Day 13 prior to the temperature increase under both PS SB and SB thermal treatments and Day 24 at the end of the experimental treatment (i.e. after corals had been exposed to 4 days with daily maximum temperatures of 34°C) (Fig. 2d). There were significant declines in endosymbiont density recorded at the end of the experiment in PS SB treated corals compared to their respective controls in both high (47%) and low (72%) (*P* < 0.001) flow treatments (Fig. 3b). No significant declines in endosymbiont density were recorded for SB treated corals at the end of the experimental period. No significant differences in endosymbiont densities were recorded within temperature treatments (Table S2).

### Experiment 2: bleaching thermal stress simulation

Over the 20-day experimental period fragments of *A. aspera* were exposed to a single thermal trajectory and average high and low flow speeds of 0.15 ± 0.004 and 0.02 ± 0.001 (see Table 2). The B thermal treatment exposed coral to an increased in temperature up to 34°C daily maxima with a ramping rate of 0.5°C for the first 10 days. Temperatures where then held with a 34°C daily maxima for 10 days, where eDHW accumulation reached 4.95°C weeks (Fig. 2g). This temperature trajectory led to bleaching levels of thermal stress, with signs of paling and mucous sloughing when exposed to daily peak temperatures. By the end of the experimental period, fragments were visually bleached but still had tissue present indicating no mortality had occurred.

#### PAM fluorometry of chlorophyll fluorescence

**Figure 4:**
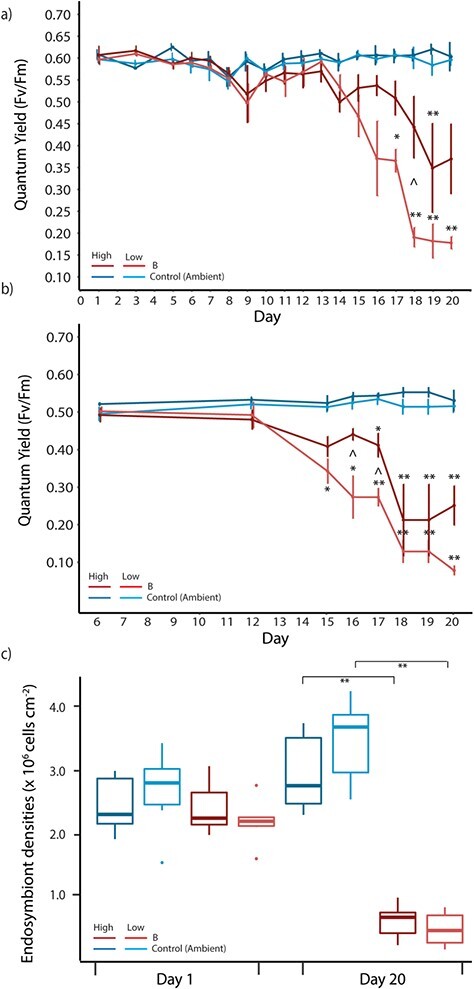
**(a)** Photophysiological measures of quantum yield during the Experiment 2: bleaching exposure stress experiment. Mean ± SE are shown for each treatment. Asterisks indicate a significant difference between the treatment and its respective control (*P < 0.05, **P < 0.01), whereas a ^ (*P* < 0.05) indicates a significant difference between heat treatment high and low flow corals**. (b)** Photophysiological measures of excitation pressure over PSII at recovery after light stress. Mean ± SE are shown for each treatment. Asterisks indicate significant difference between the treatment and its respective control (*P < 0.05, **P < 0.01), whereas a ^ (*P* < 0.05) indicates a significant difference between heat treatment high and low flow corals. Measurements begin from Day 6 and run through to the end of the experimental period (Day 20**). (c)** Endosymbiont densities on Day 1 and Day 20 of the bleaching experiment. Box plots upper and lower lines correspond to the first and third quartiles and whiskers represent the minimum and maximum values. Points indicate outliers. Asterisks indicate significant differences between heat treatments and their respective control (*P* < 0.001). On both plots, blue and red colours signify the ambient and B temperature treatment, while the darker shades within a temperature treatment represent high flow and lighter shades are low flow conditions.

Maximum quantum yield (Fv/Fm) of *A. aspera* fragments in both flow treatments decreased significantly with time under increasing thermal stress, while remaining high and relatively constant in the ambient control treatments, regardless of whether they were under high or low flow speed. The mixed effects analysis of quantum yield shows a significant three-way interaction between heat treatment, flow treatment and day (*F*_(17,423.998)_ = 9.980, *P* < 0.001), indicating effects of both temperature and flow (Table S3). Fv/Fm decreased in heat treated, low flow corals on Day 17 (*P* < 0.05) after exposure to 3.70°C weeks and remained significantly lower than controls for the remainder of the experiment (*P* < 0.001) (see Fig. 4a), while heat treated high flow corals showed declines only on Day 19 (*P* < 0.001) after exposure to 4.56°C weeks. During this two-day period, there was a single day where we see a significant difference in yields between high flow heat treated and low flow heat treated corals (Day 18, *P* < 0.05) (Fig. 4a).

This trend was also reflected in the maximum quantum yield at the end of the recovery phase of the IR curve analysis (Fig. 4b and Table S4). The mixed effects analysis of quantum yield shows a significant two-way interaction between heat treatment and day [*F*_(717.93,7)_ = 18.926, *P* < 0.001] and heat treatment and flow [*F*_(717.92,1)_ = 5.642, *P* = 0.019] (Table S4). The IR curve analysis highlighted significant declines in heat-treated low flow corals on Day 15 (*P* < 0.05), whereas heat-treated high flow corals do not show declines until Day 17 (*P* < 0.05). There were two days (16 and 17) when statistically significant differences were seen between high and low flow, heat treated corals (*P* < 0.005); high flow heat treated corals retained high yields, while low flow heat treated corals had already declined significantly (Fig. 4b).

#### Endosymbiont densities

Samples for endosymbiont densities taken on Days 1 and 20 of the experimental period showed a significant decline in high (75%) and low (72%) flow heat treated corals compared to respective controls (*P* < 0.001, Fig. 4c) (Table S5). No significant differences in endosymbiont densities were recorded within temperature treatments (Table S5).

## Discussion

Flow conditions over reefs have the potential to mediate the physiological damage and function of hard coral species under thermal stress (Nakamura and van Woesik, 2001; Nakamura *et al.*, 2003; Nakamura *et al.*, 2005; Nakamura, 2010; van Woesik *et al.*, 2012; Page *et al.*, 2019). Further understanding of the extent to which an environmental factor like flow may contribute to the potential resistance and resilience of reef areas at relevant ecological scales has wide implications for both adaptive management, as well as more novel intervention techniques. Through conducting physiological experiments and contextualizing flow treatments to conditions measured in both reef flat and slope habitats, the present study examines the impacts of high (~0.15 m s^−1^) and low (~0.03 m s^−1^) flow speeds on the physiological responses of an important reef building coral under different levels of thermal stress.

### Flow conditions over Heron Island reef

Hydrodynamic models provide information on the temporal and spatial patterns of flow conditions over a reef, but these are often limited by coarse spatial resolution (e.g. eReefs smallest resolution is 1 km grids; Steven *et al.*, 2019). Understanding the range of flow conditions at scales within which corals live, i.e. metres (individual colonies) to 10s of metres (coral beds), allows experimental conditions to be guided by an ecological context and allows for abiotic heterogeneity to be acknowledged in local reef management plans.

In the current study, flow speeds measured over the reef flat at Heron Island were generally consistent across all transects with few outliers. The high and low flow treatments used in both experiments were found to occur within the upper and lower confidence intervals across all transects on the reef flat at the time the study was undertaken (0.16 m s^−1^ and 0.093 m s^−1^). Recorded reef flat flow speeds are also representative of speeds measured over other reef flats. For example, time-average flow speeds across Kaneohe Bay Barrier Reef flat in Hawaii ranged between 0.08 and 0.22 m s^−1^ (Falter *et al.*, 2004). In the present study, measurements were taken between high and low tide to capture the full range of flow speeds experienced on the flat (Roberts and Suhayda, 1983) and in doing so we provide observations of the flow speeds to which corals may be exposed.

This daily variability in tidal flow was visible in time series recorded on the reef slope in the current study. Flow conditions on the reef slope are inherently different to those on the flat due to differences in average depth, where flow typically decreases with increasing depth (Lentz *et al.*, 2016). However, at the shallow depths where metres were deployed (in 3 m of water), it is clear that flow speeds over time are still driven by tidal patterns, wind and wave stress (Figs S3– S6). Indeed hydrodynamic circulation within coral reefs is primarily thought to be driven by wave and tidal forcing and to a lesser extent wind and buoyancy effects Monismith (2006).

### The role of water flow in a simulated sub-bleaching thermal stress event

Exposure to elevated sea-surface temperatures below the bleaching threshold has been shown to result in a number of sub-cellular and cellular responses in corals (Ainsworth *et al.*, 2008; Bonesso *et al.*, 2017), including continued declines in condition until severe bleaching is reached (PSII yield < 0.3, cell density reduction > 50%) (Ainsworth *et al.*, 2008). In this experiment, the lowest quantum yields recorded were in the SB high and low flow corals (PSII yields of ~0.4). At these values, corals showed signs of physiological damage to photosystems by the end of the experimental period but notably do not show significant differences in endosymbiont density (a proxy for bleaching severity) when compared to their respective control treatments (Fig. 3b). In contrast, endosymbiont densities recorded in corals exposed to PS SB heat treatment at the end of the experimental period were significantly lower than controls: 72% reduction in endosymbiont density was recorded in low flow corals, compared with a 47% reduction in high flow corals. Notably, these reductions in densities were not reflected in measures of photophysiology (*Fv/Fm*). Similar mismatches in declines of endosymbiont densities and photophysiology have been recorded in other coral bleaching studies (Middlebrook *et al.*, 2010; Krueger *et al.*, 2015) and could be indicative of either remaining populations of endosymbionts retaining photosynthetic efficiency or the presence of non-photophysiological impacts of thermal stress initiating expulsion (Baird *et al.*, 2009). Corals exposed to low flow and PS SB trajectory showed significantly reduced yields on the final day, compared to PS SB high flow corals that retained comparatively high yields (PSII yield of ~0.55, Fig. 3a). These results clearly indicate that exposure to high and low flow speeds does interact with potential acclamatory capacity and impacts of sub-lethal thermal stress on coral health.

Ainsworth *et al.* (2016) investigated the effect of the PS SB temperature trajectory on the stress responses of *A. aspera*. After exposure to a pre-stress pulse of 4 days with peak temperatures of 32°C, the study recorded significantly lower stress responses in pre-stressed corals compared to those exposed to only a single increase in temperature to 34°C. In the current study, significant declines were measured in endosymbiont densities in PS SB treated corals irrespective of the maintenance of higher yields. The pulse duration used in this experiment was shorter, instead temperature gradually increased to 32°C over 6 days, with a total of 2 days that reached a maximum of 32°C. Although there is evidence that this pre-stress pulse in temperature did have a beneficial effect on photosystem efficiency, the physiological acclimatory ‘signal’ may not have been strong enough to result in less severe bleaching. Equally, values of quantum yield are independent of endosymbiont density, indicating that although corals exposed to the PS SB treatment may have lower densities of endosymbionts, the populations that remain maintained high quantum yields compared to populations present in fragments exposed to a single increase in temperature (SB).

The results of this experiment suggest that corals exposed to a pre-stress pulse in temperature and high flow show an increased physiological performance compared to those under low flow conditions and/or a single bleaching trajectory. This suggests a positive interactive relationship between the protective impacts of pre-stress heating and higher flow conditions. However, the damaging effects that sub-lethal thermal stress has on the physiology of coral fragments prior to bleaching may have masked any initial beneficial effect that higher flow has on the resistance of corals to thermal stress.

### The role of water flow in a simulated bleaching thermal stress event

The temperature treatment applied in Experiment 2 reached the thermal threshold for Heron Island Reef flat (34°C) over an extended time period. A maximum daily temperature of 34°C was reached after 16 days of heat accumulation, reaching a total of 4.95°C weeks by the end of the experimental period (a total of 20 days). This meant that even though both SB (Experiment 1) and B treatments exposed corals to 4 days at 34°C, the corals exposed to B trajectory had been exposed to a greater accumulation of light and temperature stress, than those exposed to the SB trajectory. This slower ramping rate successfully allowed us to further evaluate the differential responses of corals to high and low flow under temperature stress.

Corals exposed to high and low flow speeds showed differential responses when exposed to temperatures at their thermal threshold of 34°C. Buffering effects of high flow were apparent, where high flow corals maintained a higher level of photosynthetic function when exposed to thermal stress than low flow corals. Specifically, photophysiological measurements showed differences in the efficiency, damage and recovery potential of endosymbiont PSII. There is a time offset in responses between high and low flow treated corals, where the onset of quantum yield decline was two days later in high flow heat treated corals (Day 19, eDHW = 4.56°C weeks) than low flow heat treated corals (Day 17, eDHW = 3.70°C weeks). This response is also reflected in the maximum quantum yield at the end of the recovery phase (Fig. 4b), where significant declines are recorded in heat-treated high flow corals on Day 17 and heat-treated low flow corals on Day 15. There were also 3 days (Days 16 through to 18) when differences were seen between high and low flow, heat treated corals; high flow corals retained higher yields, but low flow heat treated corals had already declined significantly. However, similarly to the sub-bleaching experiment, by the end of the experimental period both high and low flow heat treated corals showed similar declines in endosymbiont densities and therefore coral bleaching. This result indicates that any beneficial effect of high flow was short term under sustained thermal stress.

### Why might flow have this effect?

There are a number of putative mechanisms through which higher flow speeds may be able to reduce the vulnerability of a coral to thermal stress. The primary determinant of coral bleaching is generally described as the accumulation of oxidative damage caused by the production and accumulation of reactive oxygen species (ROS) produced during light and thermal stress to the endosymbiont and thermal stress in the host (Lesser *et al.*, 1990; Gates *et al.*, 1992; Lesser, 1996; Nii and Muscatine, 1997; Hoegh-Guldberg, 1999; Davy *et al.*, 2012). Eventually, the rate of damage overwhelms capacity for the host and/or endosymbiont to repair, leading to a breakdown of the symbiotic relationship. This occurs through the expulsion of the endosymbiont and/or apoptosis of the host gastrodermal cells or the endosymbionts themselves (Weis, 2008; Davy *et al.*, 2012). High flow has been hypothesized as augmenting passive diffusion of ROS away from coral tissue, limiting the amount of cellular damage that occurs (Nakamura *et al.*, 2003; Nakamura *et al.*, 2005).

Increased ROS removal has previously been described as the mechanism by which high flow lowers rates of light induced photoinhibition (Nakamura *et al.*, 2005) and enhances recovery (Nakamura *et al.*, 2003). However, ROS are volatile molecules that need to cross cell walls, membranes and tissue layers (symbiosome, gastrodermal cell wall, mesoglea and epithelial cells) before the diffusion boundary layer is reached. This represents an opportunity for damage to occur before removal from the coral tissue has taken place. The extent to which diffusion of ROS across the boundary layer is impacted by flow is yet to be explored. Furthermore, an experiment by Mass *et al.* (2010) directly looking at the effects of flow on photosynthesis under no temperature stress was unable to detect the role of a reactive species in impacting photosynthesis. Alternatively, the effect of flow could be related to an increase in flux of carbon dioxide and oxygen from the coral tissue to the surrounding water column and follow-on effects that this has on photosynthesis and dark respiration of the coral tissue.

In this study, the photosystems of endosymbionts retained higher photosynthetic efficiency in high flow, heat treated corals, despite algal cells occurring in similar reduced densities to low flow heat treated corals at the end of the experimental period. This result could be related to increased rates of mass transfer induced by thinner boundary layers under high flow conditions. During the day, the effects of flow on photosynthesis have been suggested to operate at the level of the Rubisco enzyme, a crucial protein used in the dark reaction of photosynthesis for carbon fixation (Mass *et al.*, 2010). Rubisco is capable of using both carbon dioxide and oxygen as a substrate, the former representing photosynthesis and the latter photorespiration. Photorespiration is a wasteful process when compared to photosynthesis (Ort and Baker, 2002). During the day, higher rates of photosynthetic efficiency and higher effluxes of oxygen from coral tissue have been measured in corals under high flow conditions (Finelli *et al.*, 2006; Mass *et al.*, 2010), potentially building energy reserves and maintaining a symbiotic relationship for longer. In the same way, measurements of the diffusion boundary layer near the surface of corals have revealed oxygen depletion in low flow conditions during the night (Shashar *et al.*, 1993), which in turn restricts rates of dark respiration. There is also the potential for high flow conditions to raise coral and endosymbiont respiration rates at night (Patterson *et al.*, 1991) through the increased flux of oxygen into coral tissues.

Higher rates of photosynthetic efficiency could be related to increased rates of mass transfer induced by thinner boundary layers under high flow conditions. Increased rates of mass transfer may reduce any sink limitation (i.e. photorespiration and/or electron flow) causing higher levels of photosynthetic efficiency and increased levels of respiration at night (Jones *et al.*, 1998) compared to corals under low flow. Under heat stress, this putatively allows endosymbionts of corals under high flow to maintain photosynthetic efficiency for longer and potentially increase levels of respiration and general energetic capacity for repair. This may in turn lead to greater recovery from thermal and light induced damage at night compared to corals under low flow conditions. Equally, the results of this study show that there were no differences in endosymbiont densities between high and low flow corals at the end of both experimental periods. This indicates that thermal stress accumulation throughout both experiments caused enough damage to induce a bleaching response in high and low flow corals. Endosymbiont densities were only recorded at the end of the experimental period, which means we were not able to capture timing of initial declines in density in addition to any deviation in response between high and low flow corals. An interactive effect between flow and temperature treatment on endosymbiont densities was recorded in Experiment 2, indicating that there is some effect of flow on bleaching severity. Further work is needed to uncover the relationship between flow speeds and bleaching responses under thermal stress accumulation.

There are a number of other mechanisms through which flow can impact coral function during thermal stress. The thermal boundary layer is analogous to the diffusion boundary layer, where the transfer of heat instead of the diffusion of molecules takes place. It therefore directly affects the temperature to which a coral may be exposed under lower flow conditions, where a thicker boundary layer can limit the exchange of heat transfer (Jimenez et *al.*, 2011). Under high irradiance and low flow, corals can be 0.2–0.6°C warmer than surrounding water column (Jimenez *et al.*, 2011). High flow conditions can also increase the probability of particle food capture (Sebens and Johnson, 1991). In this experiment, corals were not artificially fed but because water was taken unfiltered from the reef flat we cannot rule out the possibility of increased heterotrophic feeding of high flow corals.

Our study concentrated methodologically on impacts to endosymbiont physiology and the process of endosymbiosis breakdown in coral bleaching. Although generalizable to the coral meta-organism, further investigation is needed into the effects of flow on the coral host, its physiology and heat stress responses. Future studies should also look to quantify whether the beneficial effects of high flow are held into recovery after thermal stress has ceased and survival if thermal stress continued within the system. By the end of both experimental periods, populations of endosymbionts that remained may have been less damaged under high flow, compared to low flow, which would indicate that these fragments have the potential to recover their photosynthetic yields sooner when temperatures return to ambient conditions. Equally, if temperature stress was to continue, at some point (indicated by eventual declines in yields and endosymbiont populations) it seems that the beneficial effect of flow is no longer apparent.

### Conclusions and implications

Investigating flow patterns at the scale of metres within the context of putative beneficial physiological impacts reveals some interesting avenues for coral reef management. For example, current metres deployed on the reef slope showed that reef topography can influence local flow patterns within niche habitats on coral reefs. Variability in flow could be an ecologically relevant but overlooked phenomenon and requires further investigation. Acclimatization due to variability in historic SST trajectories has been shown to significantly impact the molecular mechanisms that underpin thermal tolerance in corals (Oliver and Palumbi, 2011; Schoepf *et al.*, 2015; Ainsworth *et al.*, 2016; Thomas *et al.*, 2018). A recent study investigating the transcriptional responses of corals during tidal fluxes suggested that variability in tide (and therefore flow) is related to acclimatory mechanisms in corals, such as ‘front loading’(Ruiz-Jones and Palumbi, 2017). This highlights a limitation of our experiment, in that corals were exposed to constant high or low flow conditions. These conditions were applied for the purpose of understanding whether high or low flow may affect coral functioning under thermal stress, but future studies should look to replicate more closely the diurnal variations in flow speed that corals experience in different reef environments.

The predictable timing of neap and spring tide times could also be used to inform management decisions proceeding predicted bleaching events. At larger reef scales, if lower flow caused by neap tides was to occur alongside cloudless days and low wind speeds, shallow reefs could be at risk of high bleaching and mortality (DeCarlo *et al.*, 2017; Burt *et al.*, 2019). Knowledge of the predominant flow direction at a site may also be able to explain local patterns of bleaching severity. At Coral Gardens (reef slope), the prevailing flow direction at the two exposed metres (1 and 2) was north-westerly, with the highest flow speeds also occurring in this direction. This same pattern has been highlighted in other studies in this region, where a north-westward flow pattern was also measured along the Capricorn Bunker group (Griffin *et al.*, 1987). These patterns also changed at each metre during neap and spring tidal phases. The morphology of coral colonies provides resistance to the natural flow of water, slowing down and causing turbulence in the water column (Reidenbach *et al.*, 2006a; Reidenbach *et al.*, 2006b; Reidenbach *et al.*, 2007). This means that under some circumstances, flow can be greatly reduced at downstream faces of a colony compared to upstream. This effect is dependent on coral morphology but has been hypothesized as causing mortality from the inner sections of branching coral colonies, which further progressed towards colony fringes during an anomalous thermal event in Iriomote, Japan (Baird *et al.*, 2018).

In conclusion, the results presented here indicate that high flow has a mediating effect on the health of endosymbiont photosystems in *A. aspera* compared to low flow conditions under both sub-bleaching and bleaching levels of thermal stress. We uncover a synergistic interaction between high flow and the protective effect of a pre-stress pulse in temperature. Measurements of physiology indicate the beneficial action of high flow on the efficiency, damage and recovery rate of endosymbiont photosystems under both direct thermal stress, and acclimatory thermal treatments. Endosymbiont photosystems retained photosynthetic efficiency in high flow heat treated corals, despite occurring in similar densities to low flow heat treated corals at the end of both experimental periods. We hypothesize that this effect may be due to increased rates of mass transfer under higher flow. However, despite a delayed onset, under sustained exposure to high levels of thermal stress corals under both high and low flow exhibit declines in photo-physiology and bleaching severity. Further work is needed to understand the effects of flow on coral host responses, in addition to coral recovery after temperature stress has subsided. Through taking a holistic approach to understanding how an environmental driver impacts coral responses to thermal stress, we have investigated the impacts an environmental factor like flow can have on the health and persistence of reefs into the future.

## Supplementary Material

Supplemental_materials_Edit_coab046
