# Supplementary material for: High flow conditions mediate damaging impacts of sub-lethal thermal stress on corals’ endosymbiotic algae
Source: Conserv Physiol. 2021 Jun 24;9(1):coab046. doi: 10.1093/conphys/coab046 (PMC8226191; doi:10.1093/conphys/coab046)
Supplement: Supplemental_materials_Edit_coab046 [file supplemental_materials_edit_coab046.pdf]

<u>**Supplemental Materials**</u>

High flow conditions mediate damaging impacts of sub-lethal thermal stress on corals' endosymbiotic algae

Page C E[1,2,3], Leggat W[3], Heron S F[4,5], Fordyce A J[3], Ainsworth T D[2]

[1]Life Sciences, Imperial College, London, SW7 2AZ, United Kingdom

[2]School of Biological, Earth and Environmental Sciences (BEES), UNSW, Kensington, NSW, 2033, Australia

[3]University of Newcastle, School of Environmental and Life Sciences, University Dr, Callaghan, NSW, 2308, Australia

[4]Physics and Marine Geophysical Laboratory, College of Science and Engineering, James Cook University, Townsville, 4811, QLD, Australia

[5]NOAA Coral Reef Watch, College Park, MD, 20740 USA

S1 Statistical analysis conducted on measured *in situ* flow conditions

The stationarity of the time series for each current meter was tested using the package tseries in R (Trapletti *et al.*, 2011) for the Augmented Dickey-Fuller Test (adf.test). A time series is stationary if its underlying statistical properties (i.e. mean, variance, autocorrelation) do not change over time. If a time series did not show stationarity, it was transformed through differencing to be made stationary (performed by subtracting the previous observation from the current observation). In addition to summary statistics for each meter, a two sample Kolmogorov-Smirnov test was used to compare the cumulative distributions of flow speeds measured at each meter using pairwise comparisons in (Team, 2019). To test whether the flow speeds measured at each meter are causal (i.e. can be used to forecast each other), patterns of flow speed were compared between loggers through testing for causality through the Granger Test using the function grangertest from the R package lmtest (Zeileis *et al.*, 2002). The test is a Wald test comparing an unrestricted model in which y is explained by the lags (up to order 1 unit of time) of y and x and the restricted model, in which y is only explained by the lags of y. Results will either confirm or reject a null hypothesis that past values of meter x do not explain values from meter y. Pairwise comparisons between each meter were conducted.

S2 Results of statistical analysis conducted on measured *in situ* flow conditions

Application of the Augmented Dickey-Fuller Test indicate that the time series data for each meter are stationary. Meters 1 and 2 have higher values in general and show similar patterns in speed over time (Figure 5a). The distributions of speeds at each meter varies (Figure 5b). Each logger showed a peak at ~ 0.10 m s^{-1} and logger three shows a bimodal distribution in speed, with peaks at both ~ 0.10 m $s^{-1}$and 0.04 m s^{-1} (Figure 5b).

Pairwise comparisons of cumulative distributions for each logger using a two sample Kolmogorov-Smirnov test indicate that all meters have significantly different distributions (1:2, D =0.137, p < 0.001; 2:3, D = 0.274, p < 0.001; 1:3, D = 0.185, p < 0.001) (see Supplemental Information, Figure S5 for a graph showing the empirical distribution function for each meter). Pairwise comparisons of meters using the granger test shows that whilst meter 1 is not able to predict meter 2 ($F_{(34557, -1)} = 0.041$, p = 0.839), nor meter 3 ($F_{(34557, -1)} = 2.338$, p = 0.127), all other meter pairs are predictable of each other (2:1, $F_{(34557, -1)} = 80.018$, p = < 0.001; 2:3, $F_{(34557, -1)} = 7.166$, p = 0.007; 3:2, $F_{(34557, -1)} = 56.662$, p = < 0.001; 3:1, $F_{(34557, -1)} = 151.52$, p = <0.001). Meter 1 and 2 show a northwesterly pattern in direction of flow over the deployment period, whilst there is no clear pattern in direction of meter 3 (Figure 5c).

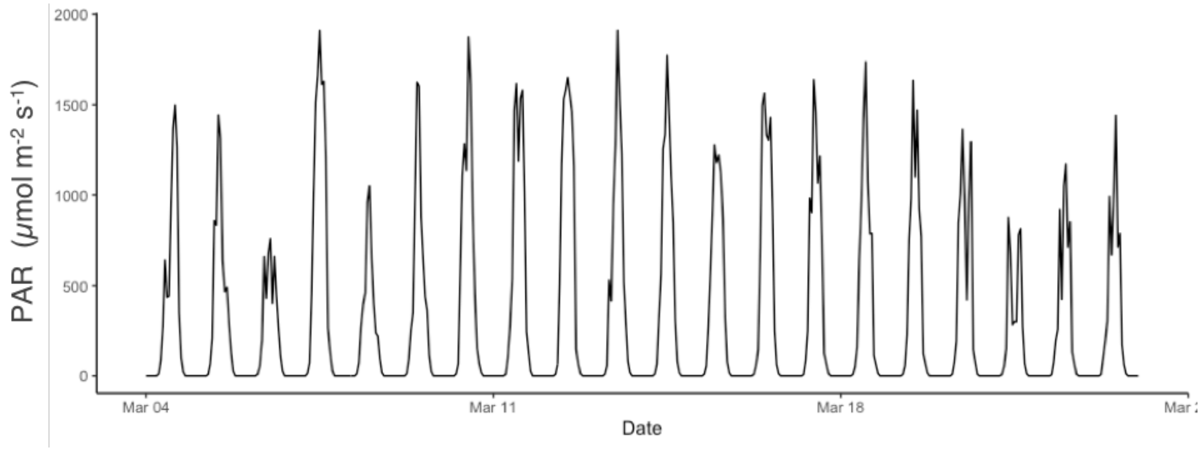

Figure S1. Average Light intensities during the bleaching level thermal stress experimental period. Light logger intensities measured across the experimental area as photosynthetically active radiation (PAR, μmol m^{-2} s^{-1}). Plot displays the hourly average across all mesocosm tanks in Experiment 2.

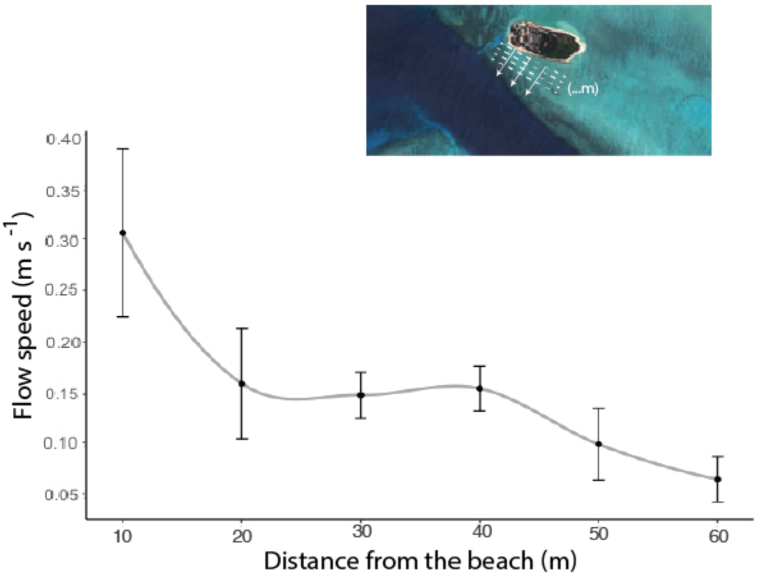

Figure S2. Flow conditions on Heron Island reef Flat. Average flow speeds across all transects at 10 m increments from the beach. Bars represent ± standard error.

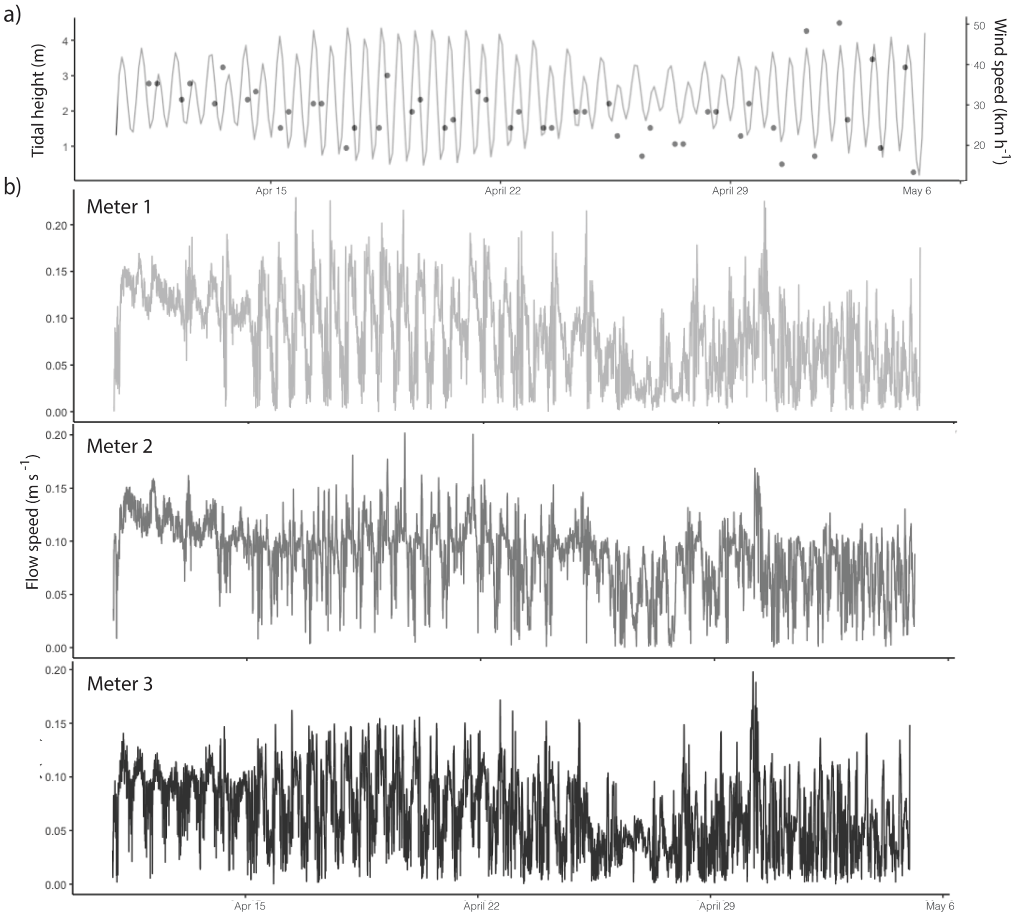

Figure S3. Flow conditions on Heron Island reef slope (Coral Gardens) a) Graph of tide height (line) and wind speed (dots) at Heron Island (10/04/2019 - 4/05/2019). Data from the Australian Government Bureau of Meteorology. **b)** Time series of water speed every minute (ms^{-1}) for meter 1, 2 and 3. **c)** A graph showing the cumulative sum flow speed (ms^{-1}) measured at current meter one, two and three coloured light, medium and dark grey respectively.

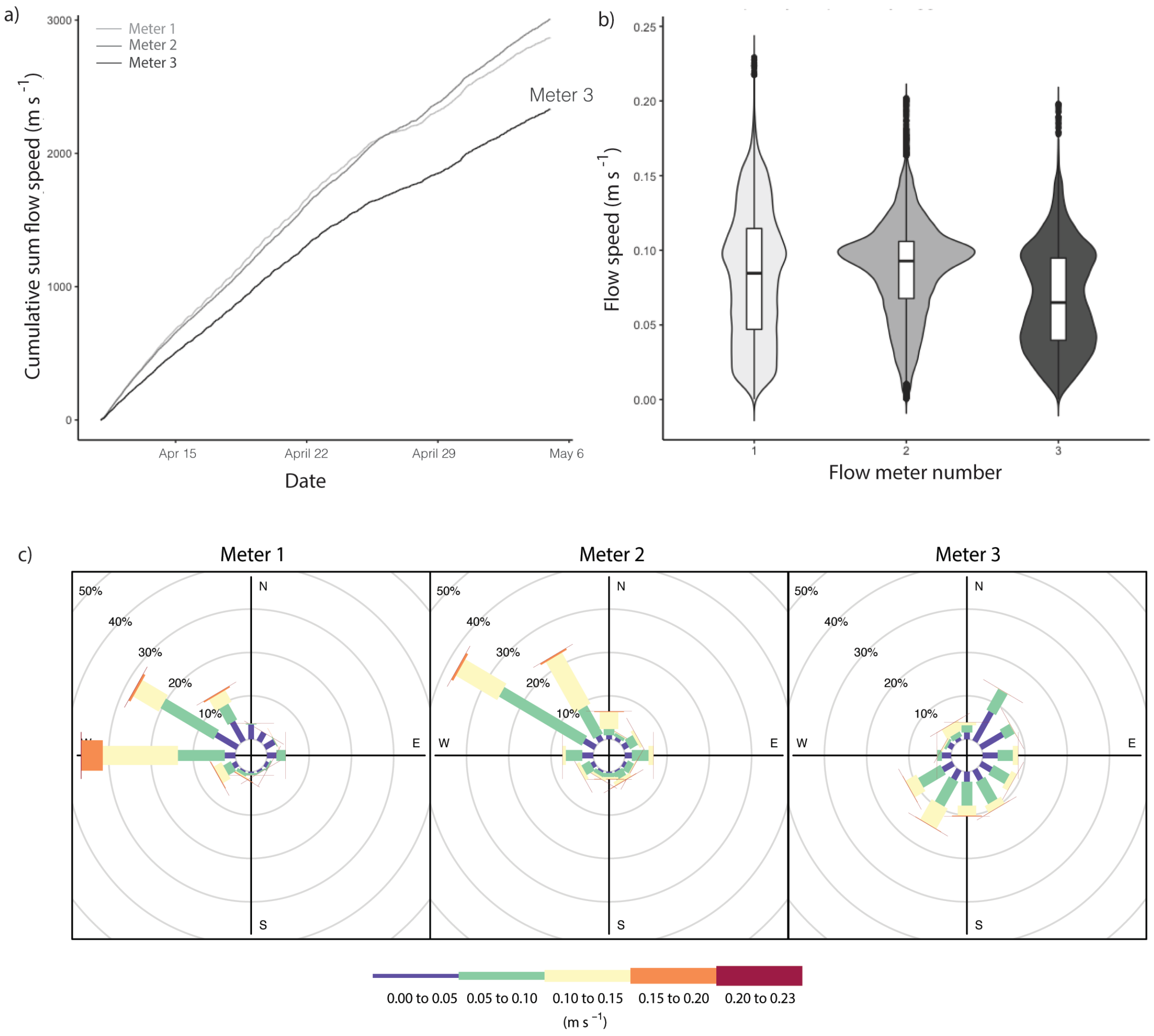

Figure S4. Flow conditions on Heron Island reef slope (Coral Gardens) a) A graph showing the cumulative sum flow speed (m s^{-1}) measured at current meters. Meters 1, 2 and 3 are coloured light, medium and dark grey respectively. **b)** Violin box plots for each meter. Boxplot shows the median value, interquartile range and upper/lower first and third quartiles. Wider sections of represent a higher probability of observation at that speed, and the thinner sections correspond to a lower probability. Points represent outliers. Meters 1, 2 and 3 are coloured light, medium and dark grey respectively. **c)** Rose plots for each meter showing the frequency (%) of different flow speeds and direction over time. Colour of bar represents speed bin.

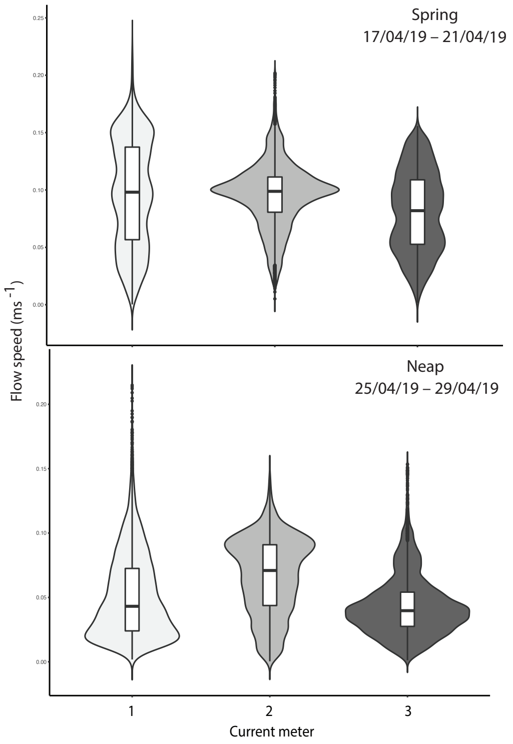

Figure S5. Violin box plots for each meter during periods defined around spring (17/04/19 – 21/04/19) and neap tides (25/04/19 – 29/04/19). Boxplot shows the median value, interquartile range and upper/lower first and third quartiles. Wider sections of represent a higher probability of observation at that speed, and the thinner sections correspond to a lower probability. Points represent outliers. Meters 1, 2 and 3 are coloured light, medium and dark grey respectively.

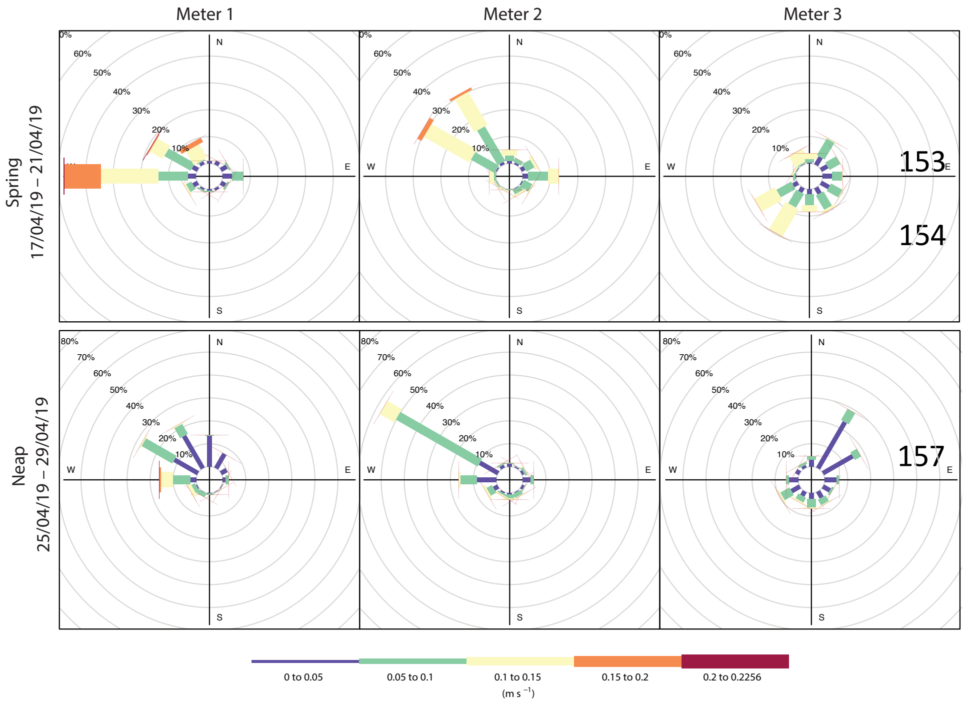

Figure S6. Rose plots for each meter showing the frequency (%) of different flow speeds and direction during periods defined around spring (17/04/19 – 21/04/19) and neap tides (25/04/19 – 29/04/19). Colour of bar represents speed bin.

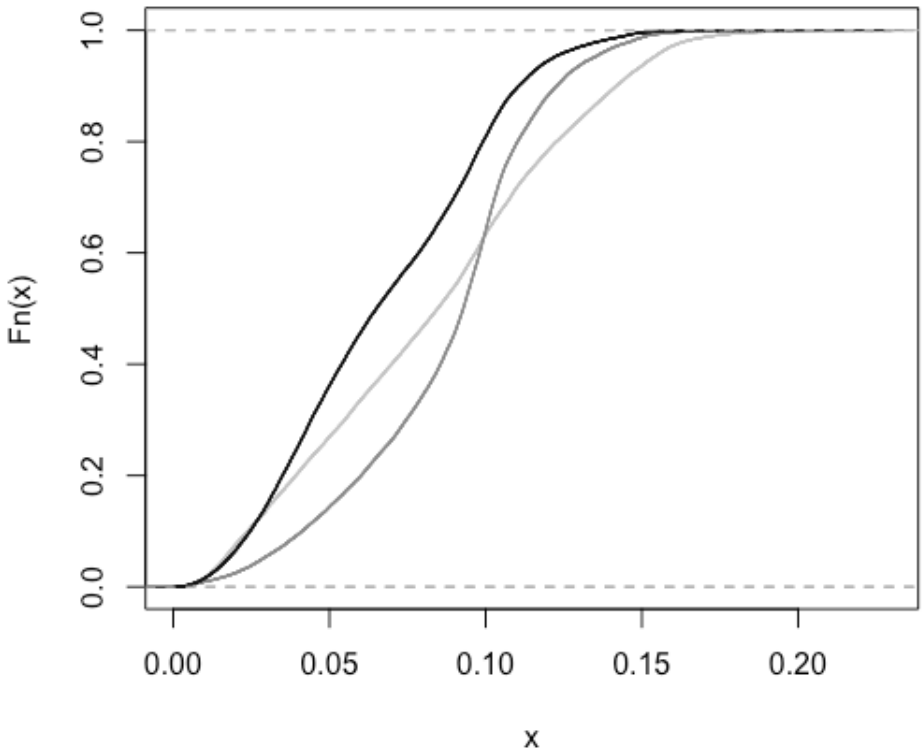

Figure S7. Graph showing the empirical distribution function (EDF) for each meter. Meter one, two and three are colored light, medium and dark grey respectively.

Table S1. Type I analysis of variance with Satterthwaite's Method from a mixed model analysis to test for the effects of day, temperature trajectory (PS SB, SB and control) and flow condition (high and low flow) on measured Fv/Fm. p-values < 0.05 are highlighted in bold. Num df = numerator degrees of freedom, den df = denominator degrees of freedom.

Effect	Num df	Den df	F-statistic	p-value
**Experiment 1: Measure of photophysiology, Quantum Yield (Fv/Fm)**				
Day	21	655	13.554	**< 0.001**
Trajectory	2	655	159.605	**< 0.001**
Flow	1	655	0.084	0.772
Day*Trajectory	42	655	4.016	**< 0.001**
Day*Flow	21	655	0.607	0.915
Trajectory*Flow	2	655	4.674	**0.010**
Day*Trajectory*Flow	42	655	0.802	0.811

Table S2. Type I analysis of variance with Satterthwaite's Method from a mixed model analysis to test for the effects of time point (Day 15 or Day 24), temperature trajectory (PS SB, SB and control) and flow condition (high and low flow) on endosymbiont densities. P-values <0.05 are highlighted in bold. Num df = numerator degrees of freedom, den df = denominator degrees of freedom.

Effect	Num df	Den df	F-statistic	p-value
**Experiment 1: Endosymbiont densities**				
Time point	1	171626778	39.922	**<0.001**
Trajectory	2	124829727	21.607	**<0.001**
Flow	1	144226759	0.199	0.655
Time point*Trajectory	2	104450442	6.059	**0.002**
Time point*Flow	1	120247022	1.181	0.276
Trajectory*Flow	2	86674254.6	0.637	0.528
Time point*Trajectory*Flow	2	76154665.8	1.489	0.225

Table S3. Type I analysis of variance with Satterthwaite's method from a mixed model analysis to test for the effects of day, temperature trajectory (B and control) and flow condition (high and low flow) on measured Fv/Fm. p-values <0.05 are highlighted in bold. Num df = numerator degrees of freedom, den df = denominator degrees of freedom.

Effect	Num df	Den df	F-statistic	p-value
**Experiment 2: Measure of photophysiology, Quantum Yield (Fv/Fm)**				
Day	17	424.007	52.096	**< 0.001**
Trajectory	1	424.005	687.934	**< 0.001**
Flow	1	423.996	87.140	**< 0.001**
Day*Trajectory	17	424.016	63.206	**< 0.001**
Day*Flow	17	423.998	8.810	**< 0.001**
Trajectory*Flow	1	423.998	47.577	**<0.001**
Day*Trajectory*Flow	17	423.998	8.408	**< 0.001**

Table S4. Type I analysis of variance table with Satterthwaite's Method from a mixed model analysis to test for the effects of day, temperature trajectory (B and control) and flow condition (high and low flow) on measured Fv/Fm after light stress and recovery of PSII (induction recovery). p-values <0.05 are highlighted in bold. Num df = numerator degrees of freedom, den df = denominator degrees of freedom.

Effect	Num df	Den df	F-statistic	p-value
**Experiment 2: Measure of photophysiology, Quantum Yield (Fv/Fm) IR curve**				
Trajectory	1	706.45	315.868	**< 0.001**
Flow	1	717.93	19.015	**< 0.001**
Day	7	717.93	10.268	**< 0.001**
Trajectory*Flow	1	717.93	5.642	**0.019**
Trajectory*Day	7	717.93	18.926	**< 0.001**
Flow*Day	7	717.93	0.694	0.676
Day*Trajectory*Flow	7	717.93	1.028	0.410

Table S5. Type I analysis of variance with Satterthwaite's Method from a mixed model analysis to test for the effects of time point (Day 1 or Day 20), temperature trajectory (B and control) and flow condition (high and low flow) on endosymbiont densities. P-values < 0.05 are highlighted in bold. Num df = numerator degrees of freedom, den df = denominator degrees of freedom.

Effect	Num df	Den df	F-statistic	p-value
		**Experiment 2: Endosymbiont densities**		
Time point	1	3.35E+40	16.891	**< 0.001**
Trajectory	1	3.35E+40	126.936	**< 0.001**
Flow	1	3.35E+40	0.2829	0.595
Time point*Trajectory	1	3.35E+40	81.386	**< 0.001**
Time point*Flow	1	3.35E+40	0.598	0.439
Trajectory*Flow	1	3.35E+40	4.246	**0.039**
Time point*Trajectory*Flow	1	3.35E+40	0.155	0.694

Table S6. Variance and standard deviation of random effects and residuals of mixed effects models conducted on response variables from Experiment 1 and Experiment 2.

Response variable	Random effect	Variance	Standard deviation
	**Experiment 1**		
Quantum Yield	Coral ID : Tank	0.0000412	0.006419
	Tank	0.0001233	0.011105
	Residual	0.0012834	0.035825
Endosymbiont density	Tank	6.12E+09	78200
	Residual	2.42E+11	491713
	**Experiment 2**		
Quantum Yield	Tank	0.0001414	0.01189
	Residual	0.0013026	0.03609
Quantum Yield: IR curve	Tank	9.77E-05	0.009882
	Residual	1.84E-02	0.135722
Endosymbiont density	Tank	0	0
	Residual	2.01E+11	448176
